# Chirality transfer from gold nanocluster to adsorbate evidenced by vibrational circular dichroism

**DOI:** 10.1038/ncomms8117

**Published:** 2015-05-11

**Authors:** Igor Dolamic, Birte Varnholt, Thomas Bürgi

**Affiliations:** 1 Département de Chimie Physique, Université de Genève, 30 Quai Ernest-Ansermet, 1211 Genève 4, Switzerland

## Abstract

The transfer of chirality from one set of molecules to another is fundamental for applications in chiral technology and has likely played a crucial role for establishing homochirality on earth. Here we show that an intrinsically chiral gold cluster can transfer its handedness to an achiral molecule adsorbed on its surface. Solutions of chiral Au_38_(2-PET)_24_ (2-PET=2-phenylethylthiolate) cluster enantiomers show strong vibrational circular dichroism (VCD) signals in vibrations of the achiral adsorbate. Density functional theory (DFT) calculations reveal that 2-PET molecules adopt a chiral conformation. Chirality transfer from the cluster to the achiral adsorbate is responsible for the preference of one of the two mirror images. Intermolecular interactions between the adsorbed molecules on the crowded cluster surface seem to play a dominant role for the phenomena. Such chirality transfer from metals to adsorbates likely plays an important role in heterogeneous enantioselective catalysis.

## Abstract (web summary)

The transfer of chirality from surfaces to molecular species may have implications in areas from the origin of homochirality to heterogeneous catalysis. Here, the authors show that a chiral gold cluster can transfer its inherent chirality to adsorbed, achiral molecules, causing them to adopt chiral conformations.

## Graphical abstract

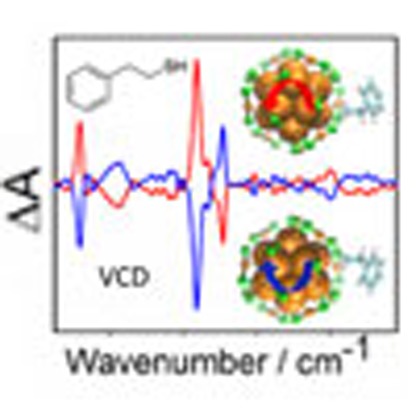

Chirality plays a prominent role in nature and has tremendous impact on biology, medicine and pharmaceutical sciences. While the origin of homochirality on earth is still unclear it is a fact that many biological macromolecules are made from chiral building blocks. In contrast, metals are highly symmetric in their bulk. However, it became clear in the last decade that the situation may change at the nanoscale^1^,^2^,^3^,^4^. In fact, metal nanoparticles or clusters covered by thiolates bear chirality as evidenced by the observation of circular dichroism (CD) activity in electronic transitions that are localized mainly in the metal part. Whetten and coworkers^5^ observed prominent CD activity for gold clusters covered by glutathione, a chiral cysteine-containing tri-peptide. Other examples of gold nanoparticles or clusters of different size and covered by different chiral thiolates were reported later^6^,^7^,^8^,^9^,^10^,^11^,^12^. Furthemore, it emerges from X-ray crystal structure studies that gold clusters are composed of a dense symmetric core covered by monomeric SR–Au–SR or dimeric SR–Au–SR–Au–SR staple motifs (SR: thiolate)^11^,^13^,^14^,^15^,^16^. Interestingly, these staple motifs are arranged in a chiral fashion on the surface of the metal core. This has been found for example in the case of Au_102_(PMBA)_44_ (PMBA=para-mercaptobenzoic acid)^13^, but also for Au_38_(2-PET)_24_ (2-PET=2-phenylethylthiolate)^14^. The Au_38_(2-PET)_24_ cluster consists of a face-fused bi-icosahedral Au_23_ core protected by six dimeric and three monomeric staple motifs. The latter are found along the equator of the prolate core, whereas the long units form two triblade fans at its ends. The two fans at the poles of the cluster have the same handedness, giving rise to chirality. We have shown before that the enantiomers of the Au_38_(2-PET)_24_ cluster, denoted C and A (clockwise/anticlockwise) for the right- and left-handed enantiomers, respectively, can be separated by chiral high-performance liquid chromatography (HPLC)^15^. Similarly, enantiomers of Au_40_(2-PET)_24_ (ref. 16) and Au_28_(TBBT)_20_ (TBBT=4-tert-butylbenzenethiolate)^11^ were separated by chiral HPLC. Furthermore, the enantiomers of the Au_102_(PMBA)_44_ were enriched by phase transfer using a chiral ammonium salt as phase transfer agent^17^.

Research on gold clusters has mainly focused on their structure and electronic properties^18^ and on the nature and reactivity of the Au–S interface^19^,^20^,^21^,^22^. Not much attention has been paid to the structure (conformation) of the adsorbed thiolates, despite its importance for the chemical behaviour of the clusters and applications in sensing or recognition^23^. One reason for this lack of knowledge is the difficulty to study the conformation of adsorbed molecules in solution. One powerful technique to probe the conformation of chiral molecules is vibrational CD (VCD)^24^, that is, the differential absorption of left- and right-circularly polarized light by a chiral sample. VCD is sensitive to the absolute configuration but also to the conformation of a chiral molecule in solution. Indeed, the technique has already been applied to study the conformation of chiral thiolates on gold particles and clusters^9^,^25^,^26^,^27^. Achiral molecules or racemic samples do not show VCD activity. Achiral molecules can adopt chiral conformations (they are transiently chiral)^28^,^29^. In the absence of a chiral environment the two enantiomeric forms of the chiral conformation are equally abundant and therefore no VCD signal can be observed.

In this contribution, we demonstrate that an achiral thiolate adsorbed on a well-defined chiral gold cluster shows significant VCD signals. The thiolates adopt a chiral conformation and for each metal cluster enantiomer, one enantiomeric form is preferred over the other. This shows that the metal cluster can transfer its chirality to the adsorbed molecule. The type of chirality transfer evidenced in this contribution is suspected to play an important role in enantioselective processes such as chiral catalysis.

## Results

### Preparation and general considerations

We have prepared Au_38_(2-PET)_24_ clusters and separated the enantiomers using chiral chromatography as described in the methods section and in more detail in the Supplementary information. Characterization of the cluster is in agreement with previous reports (see Supplementary Figs 1–3)^15^. We then measured and analysed its infrared and VCD spectra in solution.

**Figure 1:**
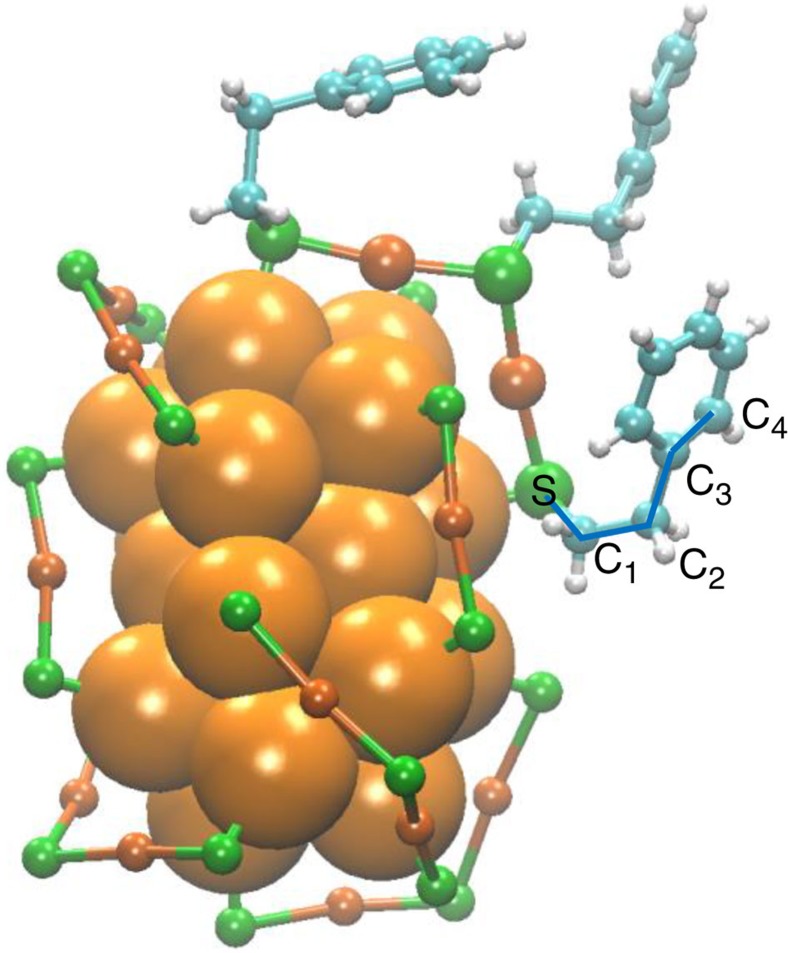
Structure of the Au_38_(2-PET)_24_ cluster. The 2-PET ligand is omitted for clarity except for one dimeric staple motif. For one 2-PET ligand the bonds relevant for the discussion of the 2-PET conformation are marked (S–C_1_–C_2_–C_3_–C_4_). Within the staple shown the centre 2-PET molecule adopts an anti conformation around the C_1_–C_2_ bond, whereas the other two molecules are in gauche conformation. Colour code: core Au atoms: orange, staple Au atoms: ochre, sulfur: green, carbon: blue, hydrogen: grey. The structure is extracted from the reported crystal structure of the cluster^14^.

The vibrational spectrum of a compound depends on its structure. In fact, VCD spectra are very sensitive to the conformation of a molecule. The conformational analysis of the entire Au_38_(2-PET)_24_ cluster in solution is complex considering the enormous number of possible conformers. Each thiolate can be oriented in two ways with respect to the staple plane (*cis* – *trans* isomerism, see also Supplementary Fig. 4). In addition, for each thiolate, several conformers can be obtained by rotation around the S–C (3 possibilities) and CH_2_–CH_2_ bonds (3 possibilities) giving rise to (2 × 3 × 3=) 18 principle conformers per adsorbed thiolate (see Fig. 1). For the whole cluster this amounts to 18^24^ conformers. Of course many of these possibilities are not feasible due to steric constraints, but a systematic conformational analysis is out of reach. To understand vibrational spectra of the cluster we therefore performed density functional theory (DFT) calculation on the free 2-phenylethylthiol and on a cyclic Au_4_(2-PET)_4_ structure, which serves as a model for the staple motifs.

### Infrared spectrum of 2-phenylethylthiol

**Figure 2:**
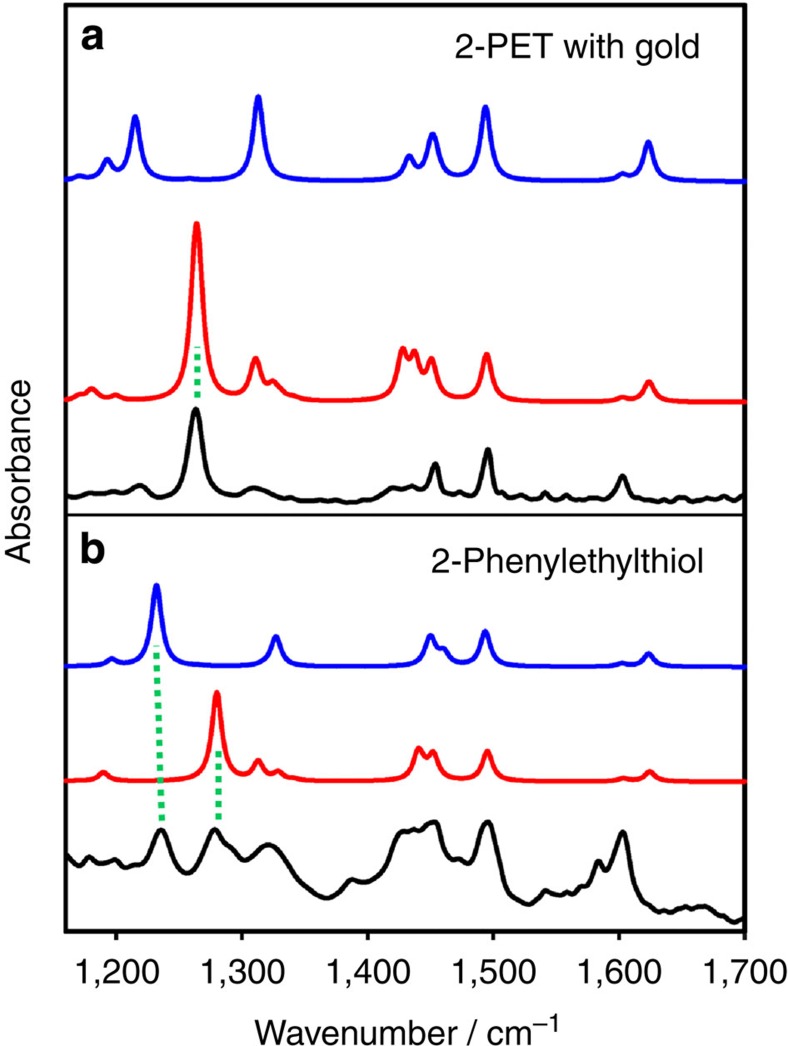
Infrared spectra of 2-phenylethylthiol and of gold cluster. Infrared spectra of 2-phenylethylthiol (**b**) and Au_38_(2-PET)_24_ cluster (**a**). The experimental spectra are shown in black. Calculated spectra are also shown (red and blue). Red (blue) spectra are associated with 2-PET and 2-phenylethylthiol, respectively, adopting gauche (anti) conformation. For the calculation of the top spectra a Au_4_(2-PET)_4_ cyclic structure was used as a model for the staple motifs found on the Au_38_(2-PET)_24_ cluster. For the red (blue) spectrum all the 2-PET molecules within the Au_4_(2-PET)_4_ structure adopt gauche (anti) conformation. Green dotted lines mark wagging modes that are sensitive to conformation.

We first consider infrared spectra of neat 2-phenylethylthiol and of the Au_38_(2-PET)_24_ cluster in Fig. 2. In the reported spectral range (1,200–1,700 cm^−1^) two principle types of vibrations are observed: Vibrations associated with the phenyl moiety (ring vibrations) and vibrations located in the CH_2_–CH_2_ part of the molecule (CH_2_ scissoring, twisting and wagging). Supported by the calculations the most prominent bands in the spectrum of neat 2-phenylethylthiol can be assigned to in-plane phenyl ring vibrations (1,603, 1,583 and 1,495 cm^−1^), CH_2_ scissoring (1,454 and 1,429 cm^−1^), phenyl in-plane C–H bending (1,322 cm^−1^) and CH_2_ wagging (1,278 cm^−1^ and 1,235 cm^−1^). A detailed assignment is given in the Supplementary Table 1. Note that the vibrations involving Au–S bonds are found below 400 cm^−1^ and are thus not accessible in the current experiment^30^.

The infrared spectra of neat 2-phenylethylthiol as well as of 2-PET within the staple depend on conformation. Figure 2 shows that the CH_2_ wagging mode is particularly sensitive to conformation. For neat 2-phenylethylthiol the calculations predict a strong mode around 1,280 cm^−1^ for the gauche conformer, whereas the corresponding mode is predicted at 1,232 cm^−1^ for the anti conformer. Comparison with the experimental spectrum (bottom black spectrum in Fig. 2) reveals that in the neat 2-phenylethylthiol liquid both conformers are abundant with bands of about equal intensity. Note that the anti conformer is slightly more stable, according to the calculations, however, this is compensated by the fact that the two gauche conformers exist (gauche+and gauche −), with positive and negative S–C_1_–C_2_–C_3_ dihedral angles, respectively (see Fig. 1). The two conformers are mirror images to each other, show identical infrared spectra and are of course equally abundant in the 2-phenylethylthiol liquid. Comparison between the calculated spectra of 2-phenylethylthiol and of Au_4_(2-PET)_4_ in Fig. 2 shows that the spectra of the free 2-phenylethylthiol and bound 2-PET are similar for the corresponding conformers (compare red and blue spectra, respectively). However, the experimental spectrum of the cluster resembles mostly the spectrum of 2-phenylethylthiol in gauche conformation, indicating that the latter plays an important role for the cluster dissolved in CD_2_Cl_2_. With this information we turn our attention to the vibrational optical activity.

### VCD spectra of Au_38_(2-PET)_24_

**Figure 3:**
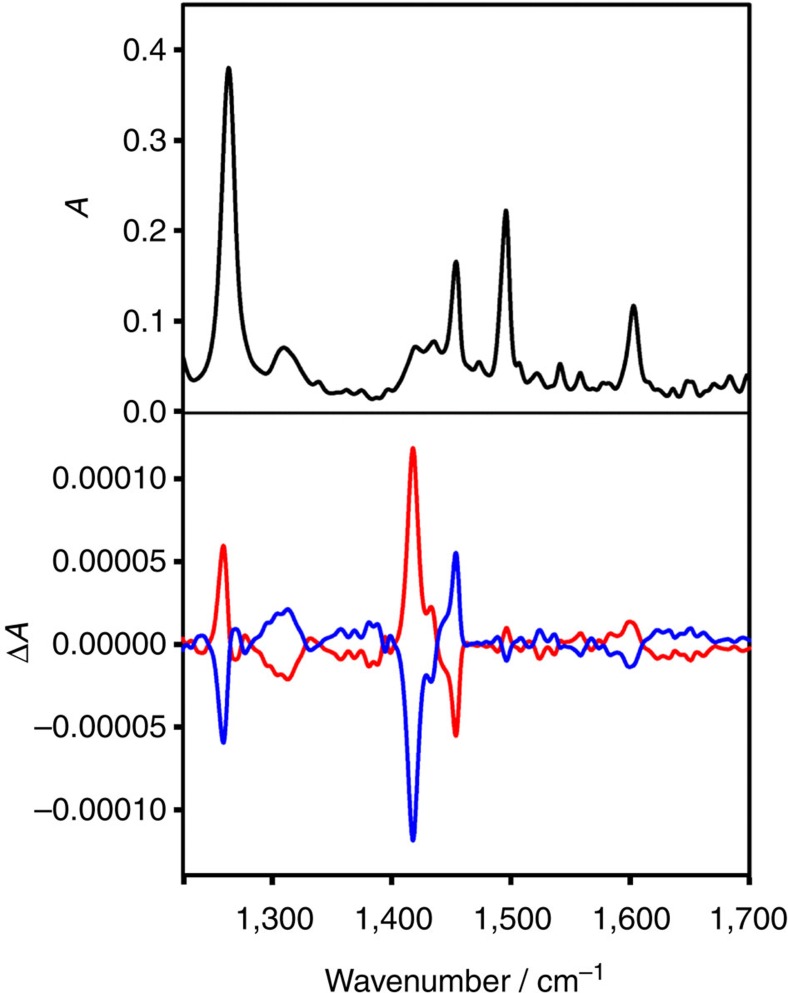
Infrared and VCD spectra of Au_38_(2-PET)_24_. Infrared spectrum of A-Au_38_(2-PET)_24_ (black) and VCD spectra of enantiomer 1 (A-Au_38_(2-PET)_24_, red) and of enantiomer 2 (C-Au_38_(2-PET)_24_, blue).

VCD spectra of the cluster enantiomers are shown in Fig. 3, together with the infrared spectrum of A-Au_38_(2-PET)_24_. The infrared spectrum of the other cluster enantiomer C-Au_38_(2-PET)_24_ is identical (see Supplementary Fig. 2). Note that the enantiomer eluting first on the HPLC (enantiomer 1) was assigned to A-Au_38_(2-PET)_24_ before, based on the comparison between experimental and calculated CD spectra^15^. The VCD spectra of A- and C-Au_38_(2-PET)_24_ show prominent bands associated with the achiral 2-PET ligand. Both the absolute strength of the VCD signals (on the order 10^−4^) and the anisotropy factors (Δ*ɛ*/*ɛ*=ΔA/A, up to ∼10^−3^) are comparable to the ones typically observed for chiral organic molecules, despite the fact that they arise from achiral 2-PET. The significant VCD signals of achiral 2-PET indicate that, once adsorbed on the chiral cluster, the molecule adopts a chiral conformation.

The intense bands in the VCD spectra of the cluster at 1,454 and 1,418 cm^−1^, respectively, are associated with CH_2_ scissoring coupled to the in-plane phenyl ring vibrations and pure CH_2_ scissoring modes, respectively. The latter has a particularly high anisotropy factor, that is, it is strong in the VCD but only weak in the infrared spectrum. In contrast, the pure in-plane phenyl ring vibration at 1,495 cm^−1^ that gives a prominent infrared signal is silent in the VCD. The very intense band at 1,263 cm^−1^ in the infrared, which shows a prominent VCD signal, is associated with a CH_2_ wagging/twisting mode, where the CH_2_ attached to the sulfur is wagging, whereas the other CH_2_ group is twisting. Note that for 2-PET in the anti conformation the corresponding modes are pure wagging modes. From the analysis it emerges that strong VCD signatures are mainly associated with the CH_2_ modes.

### Comparison with calculated spectra

**Figure 4:**
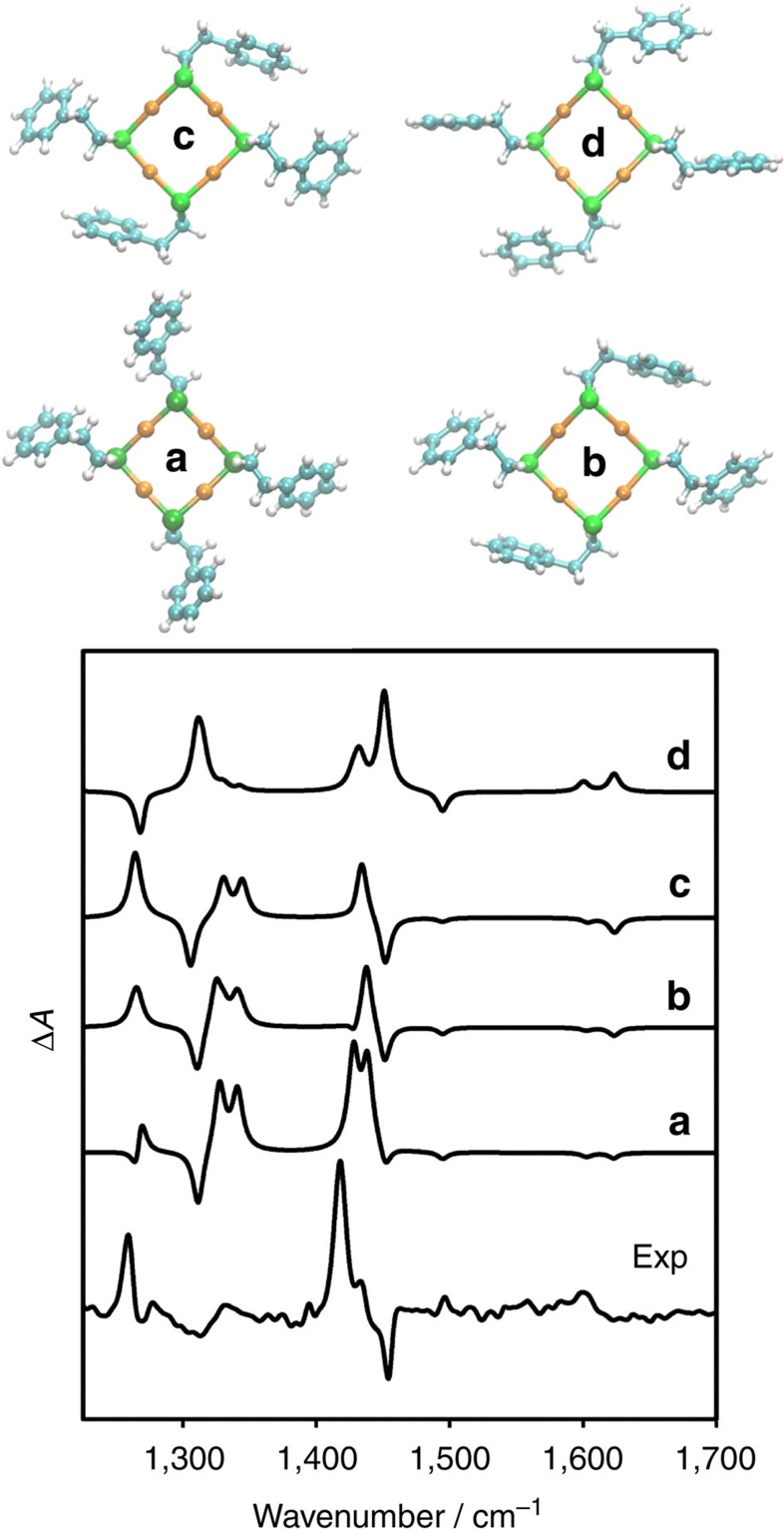
VCD spectra of calculated structures. Experimental VCD spectrum of A-Au_38_(2-PET)_24_ compared with calculated VCD spectra of Au_4_(2-PET)_4_ in different conformations (**a**–**d**). The corresponding structures are also shown. Structures **a** and **b** differ in some of the dihedral angles around the S–C bond. Structures **b**, **c** and **d** differ in the orientation of the phenyl ring (C_1_–C_2_–C_3_–C_4_ dihedral angle, see Fig. 1). See text for more information.

Using DFT calculations we have systematically investigated the effect of different conformational elements on the VCD spectra of 2-PET using the Au_4_(2-PET)_4_ model. The corresponding calculated VCD spectra are shown in Fig. 4. When all four 2-PET molecules adopt anti conformation the calculated VCD signals vanish (not shown). In contrast, when the 2-PET molecules adopt gauche conformation significant VCD signals are apparent, particularly in vibrations associated with the CH_2_ modes mentioned above. This indicates that the most important conformational degree of freedom for the VCD spectra is the dihedral angle around the CH_2_–CH_2_ bond (S–C_1_–C_2_–C_3_ dihedral angle, see definition in Fig. 1). The conformational degree of freedom around the S–C bond (dihedral angle around the S–C bond) has only a minor effect on the VCD spectra. This is underlined by a comparison between calculated VCD spectra (a) and (b) in Fig. 4, of two different Au_4_(2-PET)_4_ structures that differ in the dihedral angles around the S–C bonds. The influence of the orientation of the phenyl ring (with respect to the CH_2_–CH_2_ group, dihedral angle C_1_–C_2_–C_3_–C_4_) on the VCD spectra is more difficult to assess. In free 2-phenylethylthiol there is only one minimum on the potential energy surface of this degree of freedom and the C_1_–C_2_–C_3_–C_4_ dihedral angle (see Fig. 1) is 90° for the anti conformer and close to 100° for the gauche conformer. However, the potential energy surface in this coordinate is shallow and therefore the orientation of the phenyl ring with respect to the CH_2_–CH_2_ group is sensitive to intermolecular interactions between neighbouring 2-PET molecules, which certainly play an important role due to the high density of thiolates on the cluster surface. In our Au_4_(2-PET)_4_ model the intermolecular interactions play a less prominent role. For example in structure (b) in Fig. 4, the corresponding dihedral angles are between 100° and 110°. To investigate the influence of this structural parameter on the VCD spectra the C_1_–C_2_–C_3_–C_4_ dihedral angles were fixed at 120° (structure (c)) and 60° (structure (d)), respectively. All other coordinates were relaxed and the VCD spectra calculated. The VCD spectra of structures (b) and (c) are hardly distinguishable. For structure (d), where the phenyl ring is tilted in opposite direction with respect to the CH_2_–CH_2_ bond, the VCD spectrum changes. Particularly the weak in-plane ring vibrations slightly above 1,600 cm^−1^ and the intense wagging/twisting mode around 1,263 cm^−1^ change sign. Importantly, the pure scissoring modes around 1,418 cm^−1^ stays positive in the calculated VCD spectrum, showing that the sign of this mode depends solely on the S–C_1_–C_2_–C_3_ dihedral angle.

## Discussion

**Figure 5:**
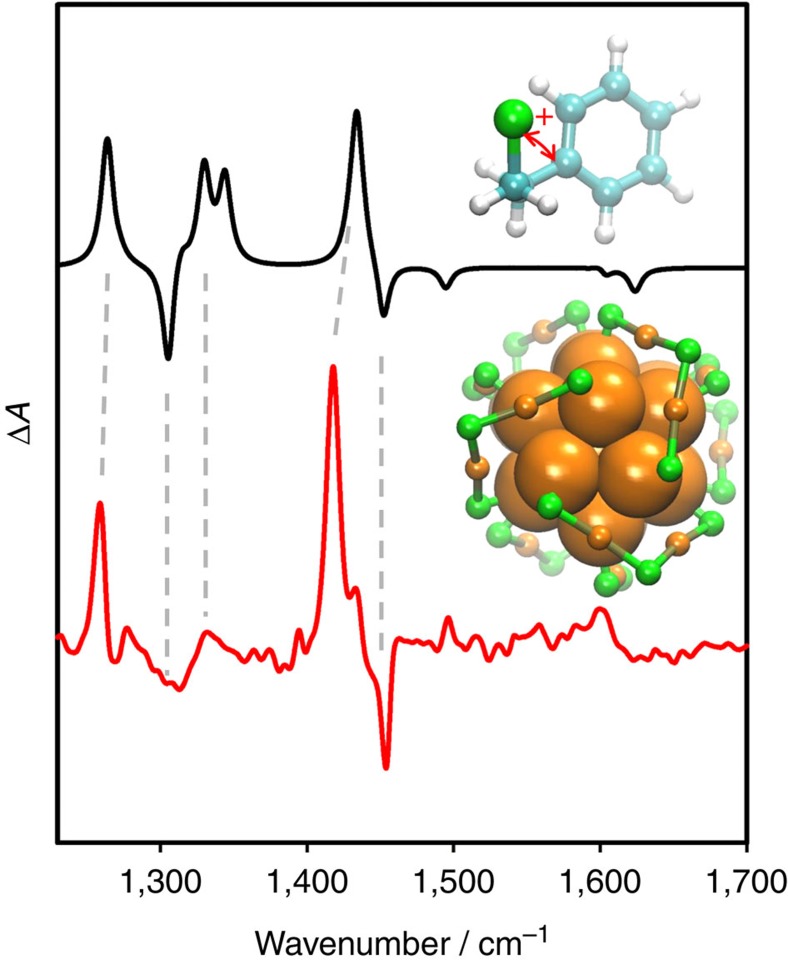
Comparison between experimental and calculated VCD spectra. Comparison between the experimental VCD spectrum of A-Au_38_(2-PET)_24_ and a spectrum that was obtained from a linear combination of calculated VCD spectra of structures (a), (c) and (d) (with relative weights 6, 75 and 19%). A structure of A-Au_38_(2-PET)_24_ is also shown (core Au atoms: orange, staple Au atoms: orange, sulfur: green, 2-PET is omitted for clarity). The preferred gauche conformation of 2-PET on A-Au_38_(2-PET)_24_ is shown at the top of the Fig. It should be noted that whereas the Au–S framework is chiral for the Au_38_(2-PET)_24_ cluster it is achiral for the Au_4_(2-PET)_4_ model used for the calculation. However, the Au–S vibrations are very low in energy and therefore large contributions from that chirality are not expected in the spectral range considered here.

Both infrared and VCD spectra of the cluster show that a significant fraction of the adsorbed 2-PET molecules adopts gauche conformation in solution. VCD furthermore clearly shows that the abundance of the two possible forms of gauche conformations (gauche+and gauche−; positive or negative S–C_1_–C_2_–C_3_ dihedral angle) is unbalanced. For the free thiol the two forms are equally abundant (and interconvert fast) and no VCD signal can be observed (the molecule is achiral). However, the VCD of the transiently chiral gauche conformers of the free thiol can be calculated (see Supplementary Fig. 5) and the spectra resemble the ones calculated for Au_4_(2-PET)_4_. Once adsorbed on the cluster the latter transfers its chirality to the molecule and one of the two gauche conformers becomes more stable than the other. Figure 5 shows qualitative good agreement between the experimental VCD spectrum of enantiomer 1 (A-Au_38_(2-PET)_24_) and a superposition of the calculated VCD spectra of structures (a), (c) and (d) (with relative weights 6, 75 and 19%). This shows that the left-handed cluster ((A-Au_38_(2-PET)_24_) induces a positive S–C_1_–C_2_–C_3_ dihedral angle (gauche +) in the 2-PET.

It is important to note that the conformational analysis done here refers to the solution structure of the cluster. In fact, the infrared spectra provide clear evidence that solution and solid-state structures are different with respect to the conformation of the 2-PET ligand. In the solution spectrum of the cluster the strong band at 1,263 cm^−1^ indicates a predominance of gauche conformers. In contrast, in the solid state of the racemic mixture strong bands at 1,220 and 1,310 cm^−1^ indicate a much larger fraction of anti conformers (Supplementary Fig. 6). Interestingly, in the solid of the enantiopure sample the fraction of gauche to anti conformers is again different (Supplementary Fig. 6). This shows that packing forces in the solid-state influence the conformation of the 2-PET ligand.

In summary, VCD measurements reveal a chirality transfer from an intrinsically chiral metal particle or cluster to the adsorbed achiral molecules. This is the first direct observation of chirality transfer of this kind. Chirality transfer via hydrogen bonding and other intermolecular interactions has been evidenced before^31^,^32^ and is an issue in many important fields of modern chemistry ranging from supramolecular chemistry^33^ to catalysis^34^. Note that in vibrational optical activity the term ‘chirality transfer' is used if an achiral structure shows optical activity due to its interaction with a chiral environment^31^,^32^. The optical activity observed in our case arises due to a mirror symmetry breaking, which favours one enantiomer of a transiently chiral molecule. Interestingly, in coordination chemistry organic ligands with defined configuration are used to transfer the chiral information to the metal centre^35^. In the system studied here it is in contrast the metal particle that transfers its chirality to the organic molecule.

## Methods

### Isolation, characterization and enantioseparation of Au_38_(2-PET)_24_

The Au_38_(2-PET)_24_ cluster was prepared and purified according to previously reported protocols^36^,^37^. In brief, tetrachloroauric acid and L-glutathione were dissolved in acetone and reduced by sodium borohydride. The resulting clusters were then dissolved in water and a mixture of ethanol and toluene and 2-phenylethylthiol was added. Heating to 80 °C gave a mixture of Au_n_(2-PET)_*m*_ (*n*: 25–144, *m*=18–60) containing Au_38_(2-PET)_24_ as major cluster. Excess thiol was removed by extensive methanol washing and the crude clusters were size-selected by size exclusion chromatography. For more details see Supplementary Methods.

The pure Au_38_(2-PET)_24_ clusters were characterized by matrix-assisted laser desorption/ionization mass spectrometry and ultraviolet–visible spectroscopy (see Supplementary Fig. 1), which showed the characteristic mass and optical transitions of the cluster. Enantiomers of the cluster were then separated on a chiral cellulose-based semi-preparative column using *n*-hexane/*i*-propanol (80/20) as eluent. The two enantiomers of the cluster were collected in several HPLC runs. CD spectra of the separated clusters (Supplementary Fig. 3) are identical to the ones reported before^15^.

### Infrared and VCD of Au_38_(2-PET)_24_

For infrared and VCD experiments the clusters (anticlockwise and clockwise versions of Au_38_(2-PET)_24_, respectively) were dissolved in CD_2_Cl_2_ at a concentration of ∼10 mg in 180 microliter and measured in a sealed transmission cell equipped with CaF_2_ windows and 200 μm metal spacer at room temperature. For comparison the infrared spectrum of neat 2-phenylethylthiol was also measured in a sealed NaCl cell. Infrared and VCD spectra of the cluster were recorded on a Bruker PMA 50 accessory coupled to a Tensor 27 Fourier transform infrared spectrometer.

### DFT calculations

The geometry optimizations, vibrational frequencies, infrared absorption and VCD intensities were performed by DFT. For the gold atoms an effective core potential was used. The calculations were performed using the b3pw91 functional and a LanL2DZ basis set for Au and a 6-31G(d,p) basis set for all other atoms.

## Author contributions

B.V. prepared the clusters. I.D. separated the clusters and performed the VCD measurement. T.B. designed the concept of the work and performed the calculations. All the authors contributed to the writing of the manuscript. The project was supervised by T.B.

## Additional Information

**How to cite this article**: Dolamic, I. *et al*. Chirality transfer from gold nanocluster to adsorbate evidenced by vibrational circular dichroism. *Nat. Commun*. 6:7117 doi: 10.1038/ncomms8117 (2015).

## Supplementary Material

Supplementary Information Supplementary Figures 1-6, Supplementary Table 1, Supplementary Methods and Supplementary References
