# Supplementary material for: Chirality transfer from gold nanocluster to adsorbate evidenced by vibrational circular dichroism
Source: Nat Commun. 2015 May 11;6:7117. doi: 10.1038/ncomms8117 (PMC4432620; doi:10.1038/ncomms8117)
Supplement: Supplementary Information — Supplementary Figures 1-6, Supplementary Table 1, Supplementary Methods and Supplementary References [file ncomms8117-s1.pdf]

Supplementary Figures

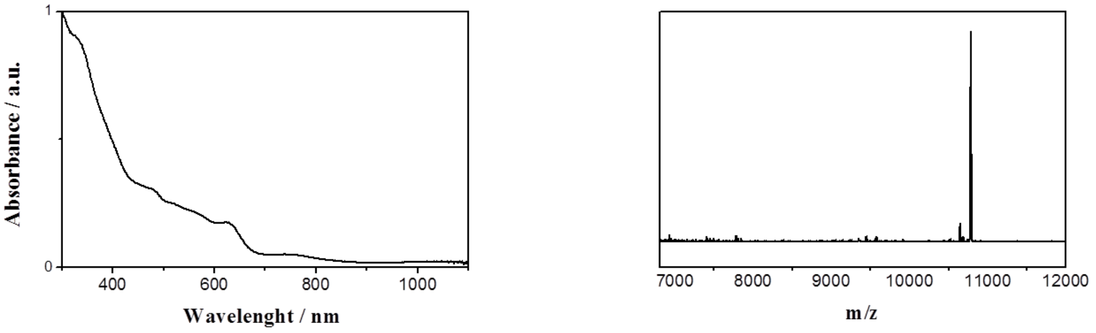

Supplementary Figure 1. Characterization of racemic Au_{38}(2-PET)$_{24}$. Left: UV-vis spectrum of *rac*-Au_{38}(2-PET)$_{24}$. Right: MALDI mass spectrum of rac-Au_{38}(2-PET)$_{24}$.

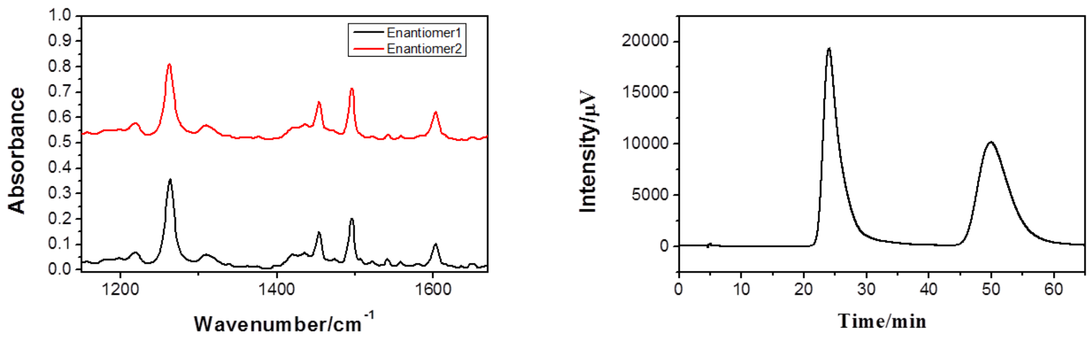

Supplementary Figure 2. Characterization of enantiomers of Au_{38}(2-PET)$_{24}$. Left: Infrared spectra of Au_{38}(2-PET)$_{24}$ enantiomers, E1 (black) and E2 (red). Right: HPLC chromatogram of Au_{38}(2-PET)$_{24}$ enantiomers separation.

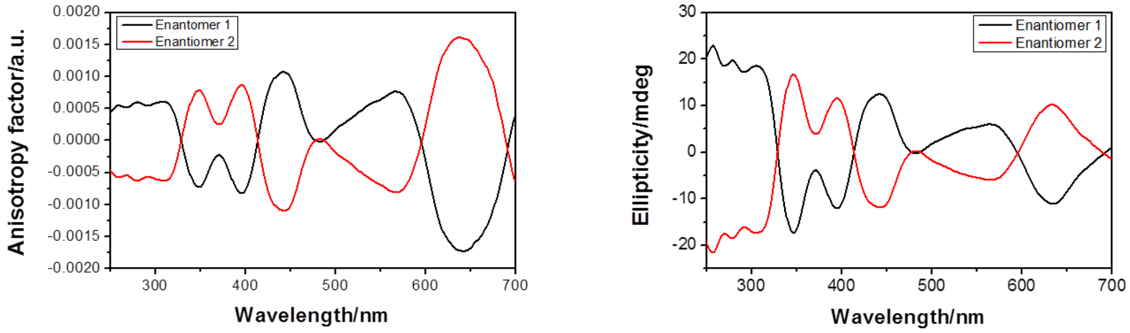

Supplementary Figure 3. Chiroptical properties of Au_{38}(2-PET)$_{24}$. Left: Anisotropy factor of enantiomers E1 (black) and E2 (red). Right: CD spectra of collected enantiomers, E1 (black) and E2 (red).

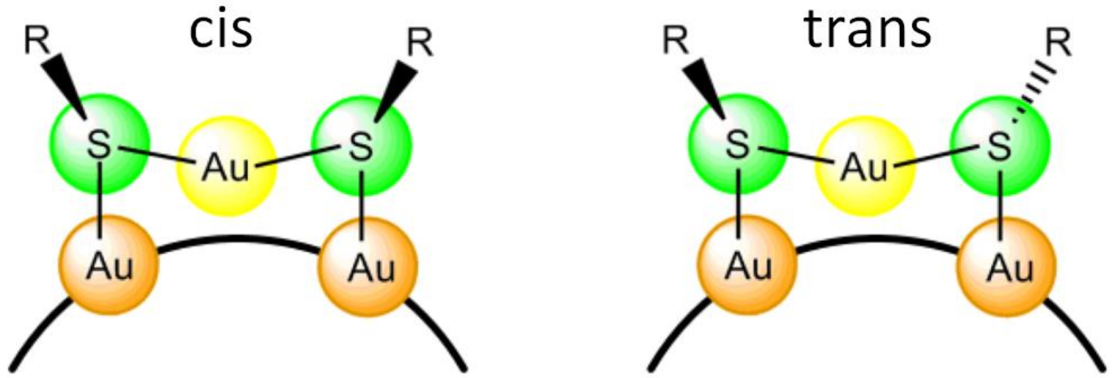

Supplementary Figure 4. Illustration of cis – trans isomerism in staple motifs. Left: cis isomer, right: trans isomer.

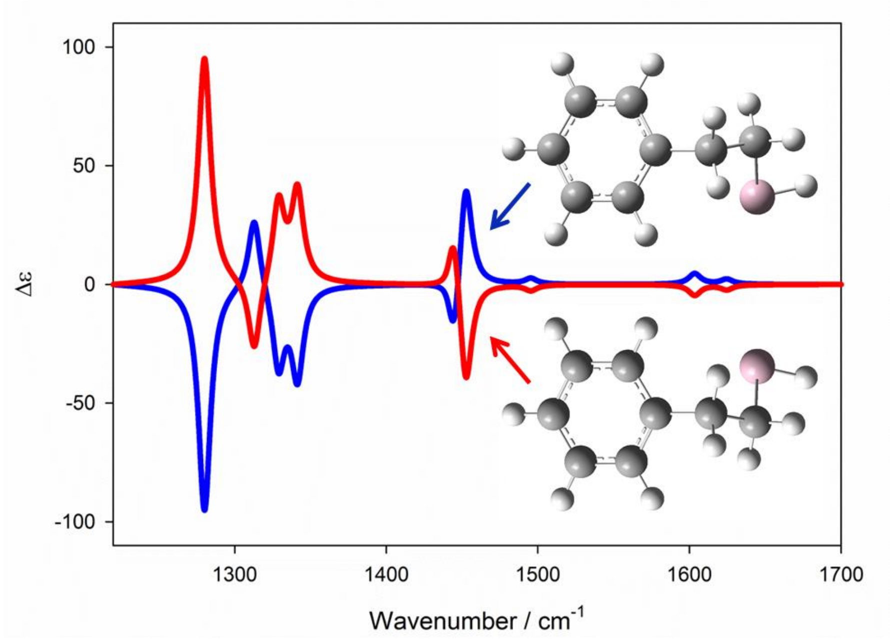

Supplementary Figure 5. Calculated vibrational circular dichroism (VCD) spectra of 2-phenylethylthiol. Left: Calculated VCD spectra for the (transiently) chiral gauche conformations of free 2-phenylethylthiol.

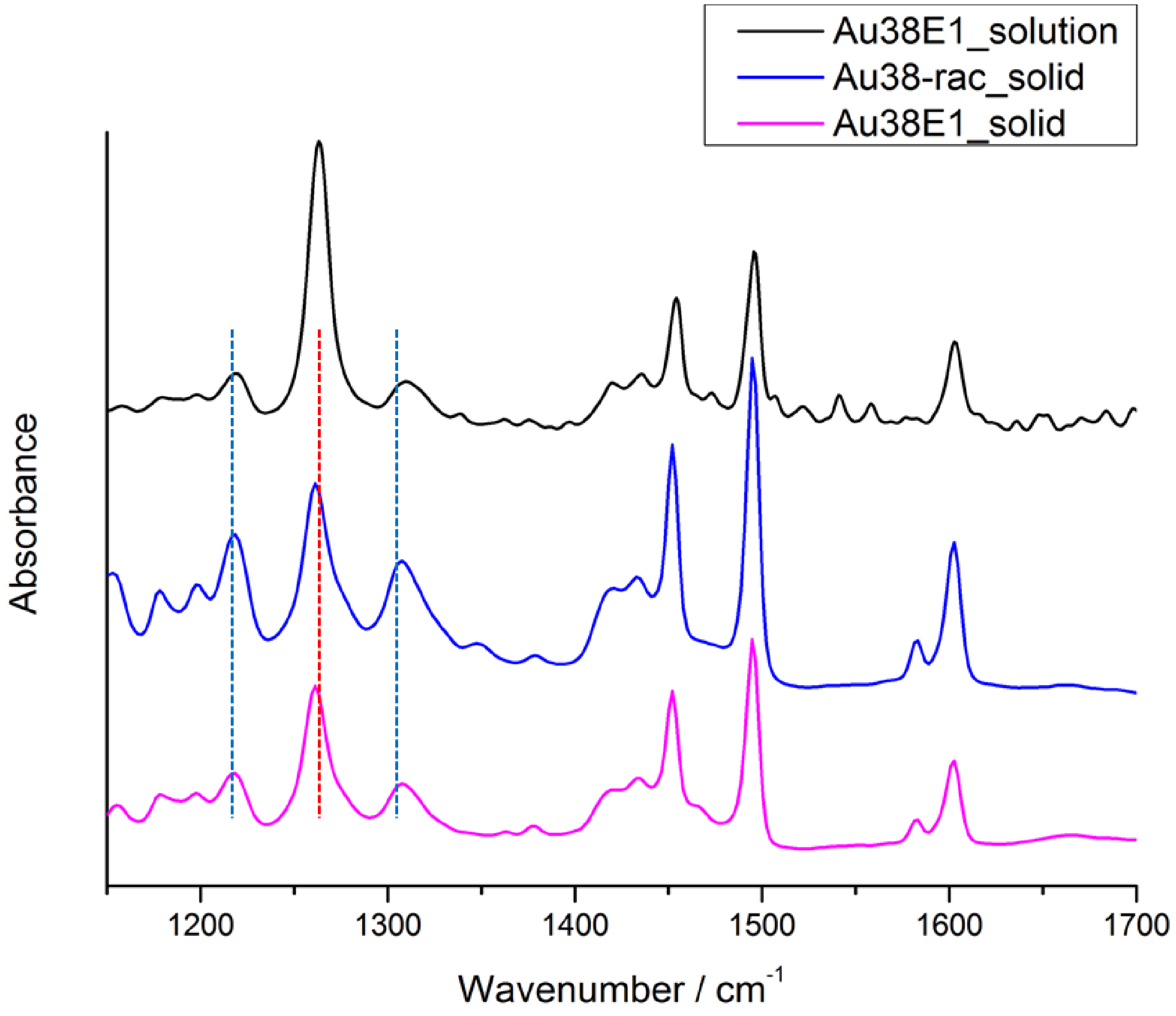

Supplementary Figure 6. Infrared spectra of Au_{38}(2-PET)$_{24}$ in different states. Black: dissolved in CD_2Cl_2 (enantiomer 1), blue: solid of racemic mixture, pink: solid enantiomer 1. The marked CH_2 bands are associated with the 2-PET ligand in anti (blue) and gauche (red) conformation, respectively.

Supplementary Tables

Supplementary Table 1: Experimental and calculated bands of selected vibrations of 2-phenylethylthiol, $Au_4(2\text{-PET})_4$ and $Au_{38}(2\text{-PET})_{24}$.Values are given in wavenumbers (cm^{-1}).

vibration	2-PET, thiol (g), calc	2-PET, thiol (a), calc	$Au_4(2\text{-PET})_4$ calc[a]	2-PET, thiol exp	$Au_{38}(2\text{-PET})_{24}$ exp
Ring, IP	1625	1623	1624	1603	
Ring, IP	1603	1602	1602	1583	1602
Ring, IP	1496	1494	1495	1495	1495
CH_2 scissoring; Ring, IP	1452		1451		1454
CH_2 scissoring	1444	1449	1438	1454	
CH_2 scissoring	1440	1461	1428	1429	1420
Ring, IP	1342				
Ring, IP; CH_2 wag;	1329	1327	1331	1322	1321
CH_2 wag; Ring, IP	1313		1311		1309
CH_2 wag; CH_2 twist[b]	1280	1232	1266	1278, 1235	1263
CH_2 twist	1190	1196	1183	1199	
CH_2 twist	1127	1122	1125	1178	
CH_2 twist; Ring, IP	1076	1071	1074	1075	
Ring, IP	1030	1028	1030		
νC-C, C-S-H bend	1009	1048	998	1029	

IP: in plane; g: gauche; a: anti; [a] structure b in Figure 4 of manuscript; [b] pure wagging mode for anti conformation.

Supplementary Methods

I Synthesis and Size-Selection of rac-$Au_{38}(2\text{-}PET)_{24}$

General Remarks. Tetrachloroauric acid trihydrate (Aldrich, 99.9+ %), reduced *L*-glutathione (Sigma-Aldrich, > 99 %), sodium borohydride (Fluka, > 96 %), 2-phenylethylthiol (Aldrich, 98 %), 3-(4-*tert*-Butylphenyl)-2-methyl-2-propenylidene]malononitrile (Aldrich, > 98 %), methanol (VWR, > 99.8 %), acetone (Fluka, > 99.5 %), methylene chloride (Sigma-Aldrich, > 99.9 %), toluene (Fisher Chemical, > 99.8 %), hexane (Sigma-Aldrich, HPLC grade), isopropanol (Sigma-Aldrich, HPLC grade), regenerated cellulose membranes (0.2 µm, Sartorius), PTFE syringe filters (0.2 µm, Carl Roth) and Bio Beads SX-1 (BioRad) were used as received if not mentioned otherwise. Milli-Q water (> 18 MΩ) was used.

Preparation and Isolation of $Au_{38}(2\text{-}PET)_{24}$. *Step 1 – Synthesis of L-glutathionate protected clusters, $Au_n(SG)_m$.* Tetrachloroauric acid trihydrate (2.54 mmol) and *L*-glutathione (10.18 mmol) were mixed in acetone (20ml) at room temperature and stirred for around 20 minutes. A yellow cloudy suspension was formed and cooled to $0^{o}C$ in an ice bath. A freshly prepared ice-cooled solution of sodium borohydride (30 mmol) in Milli-Q water (30 mL) was rapidly added to the suspension under vigorous stirring. The formation of Au nanoclusters was observed by a color change of the suspension to black. After 20 minutes aging of the $Au_n(SG)_m$ clusters, the clear acetone supernatant was decanted and around 10 ml of Milli-Q water was added to dissolve the clusters.

Step 2 – Thermal etching towards Au_{38} (2-PET)$_{24}$. Water solution of L-glutathione nanoclusters from step 1(500-600mg of $Au_n(GS)_m$ in 10ml of Milli-Q water) was mixed with

ethanol (0.5 ml), toluene (4 ml) and 2-phenylethylthiol (4 ml). The solution was heated to 80 ^{o}C and thermal etching was allowed to continue for 5 hours under air atmosphere. The organic phase was washed with methanol several times to remove the excess of the added thiol and other by products and filtered over a regenerated cellulose filter (0.2 µm).

Step 3 – Size-selection of rac-Au_{38}(2-PET)$_{24}$. 45 g of BioRad BioBeads SX-1 were suspended in about seven times the bed volume of toluene. The beads were allowed to swell overnight and given into a glass column (100 cm in length and 2.5 cm in diameter) equipped with a glass frit (G4). The crude clusters from step 2 were dissolved in a minimum amount of toluene and repeatedly eluted using toluene as mobile phase until the eluting clusters were purely composed of Au_{38}(2-PET)$_{24}$. The collected fractions were characterized by UV-vis spectroscopy until no further change was observed. The fraction identified as *rac*-Au_{38}(2-PET)$_{24}$ was washed with methanol and passed over a PTFE syringe filter, as described in step 2, before characterization.

II Characterization of Au_{38}(2-PET)$_{24}$

UV-vis spectroscopy. UV-vis spectra were recorded on a *Varian Cary 50* spectrophotometer using a quartz cuvette of 10 mm pathlength. Spectra were measured in methylene chloride and normalized at 300 nm.

MALDI analysis. Mass spectra were obtained using a mass spectrometer Shimadzu Biotech Axima in positive linear mode. 3-(4-*tert*-Butylphenyl)-2-methyl-2-propenylidene]malononitrile (DCTB) was used as the matrix with a 1:1000 analyte : matrix ratio. A volume of 1 µL of the analyte/matrix mixture was applied to the target and air-dried.

HPLC separation of enantiomers. Chromatographic separation of the enantiomers was achieved on a *JASCO 20XX* HPLC system equipped with a *Phenomenex Lux-Cellulose-1* (5 µm, 250 x 10 mm) column. For detection, a *JASCO 2070plus* UV-vis detector was used. Pathlength was 10 mm and the wavelength was set to 380 nm. The analytes were eluted at a flow rate of 3 mL/min using n-hexane : iso-propanol (80 : 20) at room temperature. Racemic $Au_{38}(2\text{-PET})_{24}$ clusters were separated and collected over several runs. The collected enantiomers were additionally washed with methanol and cleaned by passing them through size exclusion column to remove any possible impurities present in the sample.

CD spectroscopy. For the measurements of the CD spectra, the clusters were dissolved in dichloromethane and measured in a 10 mm cuvette on a JASCO J-815 spectrometer. The anisotropy factor *g* was calculated using the simultaneously measured UV-vis spectra. All measurements were performed at 20°C.

IR and VCD spectroscopy. IR and vibrational circular dichroism (VCD) spectra of the cluster were recorded on a Bruker PMA 50 accessory coupled to a Tensor 27 Fourier transform infrared spectrometer. A photoelastic modulator (Hinds PEM 90) set at l/4 retardation was used to modulate the handedness of the circular polarized light. Demodulation was performed by a lock-in amplifier (SR830 DSP). An optical low-pass filter (< 1800 cm^{-1}) in front of the photoelastic modulator was used to enhance the signal/noise ratio. Spectra were recorded with a sealed transmission cell equipped with CaF_2 windows and a 200 µm metal spacer. Solutions of *A*- and *C*-$Au_{38}(2\text{-PET})_{24}$, respectively, in CD_2Cl_2 at concentrations of about 10 mg in180 µl were used. The measurements of the two solutions were done under identical conditions and the spectra were subtracted from each other to eliminate artefacts.

Both sample and reference were measured at a resolution of 4 cm^{-1} for ten hours in time slices of one hour, corresponding to about 42600 scans in total for each sample. Spectra are presented without further data processing.

III DFT calculations

Calculations were performed on the isolated 2-PET as well as on a Au_4(2-PET)$_4$ model. The vibrational frequencies, IR absorption and VCD intensities were calculated with Density Functional Theory (DFT) for geometry-optimized structures. For the gold atoms an effective core potential was used. The calculations were performed using the b3pw91 functional and a LanL2DZ basis set for Au and a 6-31G(d,p) basis set for all other atoms. Frequencies were scaled by a factor of 0.97. IR absorption and VCD spectra were constructed from calculated dipole and rotational strengths assuming Lorentzian band shape with a half-width at half maximum of 5 cm^{-1}. All calculations were performed using Gaussian09, Revision C.01.[1]

Supplementary References

1) Gaussian 09, Revision C.01, M. J. Frisch, G. W. Trucks, H. B. Schlegel, G. E. Scuseria, M. A. Robb, J. R. Cheeseman, G. Scalmani, V. Barone, B. Mennucci, G. A. Petersson, H. Nakatsuji, M. Caricato, X. Li, H. P. Hratchian, A. F. Izmaylov, J. Bloino, G. Zheng, J. L. Sonnenberg, M. Hada, M. Ehara, K. Toyota, R. Fukuda, J. Hasegawa, M. Ishida, T. Nakajima, Y. Honda, O. Kitao, H. Nakai, T. Vreven, J. A. Montgomery, Jr., J. E. Peralta, F. Ogliaro, M. Bearpark, J. J. Heyd, E. Brothers, K. N. Kudin, V. N. Staroverov, T. Keith, R. Kobayashi, J. Normand, K. Raghavachari, A. Rendell, J. C. Burant, S. S. Iyengar, J. Tomasi, M. Cossi, N. Rega, J. M. Millam, M. Klene, J. E. Knox, J. B. Cross, V. Bakken, C. Adamo, J. Jaramillo, R.

Gomperts, R. E. Stratmann, O. Yazyev, A. J. Austin, R. Cammi, C. Pomelli, J. W. Ochterski, R. L. Martin, K. Morokuma, V. G. Zakrzewski, G. A. Voth, P. Salvador, J. J. Dannenberg, S. Dapprich, A. D. Daniels, O. Farkas, J. B. Foresman, J. V. Ortiz, J. Cioslowski, and D. J. Fox, Gaussian, Inc., Wallingford CT, 2010.
